# Biodegradable Innovations: Harnessing Agriculture for Eco-Friendly Plastics

**DOI:** 10.3390/jox16010008

**Published:** 2026-01-06

**Authors:** Komal Pandey, Baljeet Singh Saharan, Yogender Singh, Pardeep Kumar Sadh, Joginder Singh Duhan, Dilfuza Jabborova

**Affiliations:** 1 Department of Microbiology, Chaudhary Charan Singh Haryana Agricultural University, Hisar 125004, India; pandeykomal444@gmail.com; 2 Department of Environmental Studies, Shivaji College, University of Delhi, New Delhi 110027, India; 3 Department of Biotechnology, Chaudhary Devi Lal University, Sirsa 125055, India; pardeep.sadh@gmail.com; 4 Department of Biotechnology, Graphic Era (Deemed To Be University), Dehradun 248002, India; 5 Institute of Genetics and Plant Experimental Biology, Uzbekistan Academy of Sciences, Kibray 111208, Uzbekistan; dilfuzajabborova@yahoo.com

**Keywords:** bioplastics, sustainability, biodegradability, plastic pollution, agricultural biopolymers

## Abstract

Agricultural biomass has potential as a renewable and versatile carbon feedstock for developing eco-friendly and biodegradable polymers capable of replacing conventional petrochemical plastics. To address the growing environmental concerns associated with plastic waste and carbon emissions, lignocellulosic residues, edible crop by-products, and algal biomass were utilized as sustainable raw materials. These biomasses provided carbohydrate-, lipid-, and lignin-rich fractions that were deconstructed through optimised physical, chemical, and enzymatic pretreatments to yield fermentable intermediates, such as reducing sugars, organic acids, and fatty acids. The intermediates were subsequently converted through tailored microbial fermentation processes into biopolymer precursors, primarily polyhydroxyalkanoates (PHAs) and lactate-based monomers. The resulting monomers underwent polymerization via polycondensation and ring-opening reactions to produce high-performance biodegradable plastics with tunable structural and mechanical properties. Additionally, the direct extraction and modification of naturally occurring polymers, such as starch, cellulose, and lignin, were explored to develop blended and functionalized bioplastic formulations. Comparative evaluation revealed that these biomass-derived polymers possess favourable physical strength, thermal stability, and biodegradability under composting conditions. Life-cycle evaluation further indicated a significant reduction in greenhouse gas emissions and improved carbon recycling compared to fossil-derived counterparts. The study demonstrates that integrating agricultural residues into bioplastic production not only enhances waste valorization and rural bioeconomy but also supports sustainable material innovation for packaging, farming, and consumer goods industries. These findings position agriculture-based biodegradable polymers as a critical component of circular bioeconomy strategies, contributing to reduced plastic pollution and improved environmental sustainability.

## 1. Introduction

Plastic pollution has emerged as a defining anthropogenic stressor of the twenty-first century, with persistent polymers now detected in agricultural soils, marine food webs, and even human tissues. Conventional plastics are produced almost entirely from fossil carbon, degrade extremely slowly, and fragment into microplastics that accumulate across trophic levels, where they can induce oxidative stress, inflammation, and potential carcinogenic effects. Meanwhile, their production and incineration intensify greenhouse gas emissions and resource depletion. In response, attention has shifted toward bioplastics materials derived partly or wholly from renewable biomass, and in some cases, designed for controlled biodegradation as a key enabler of a circular and climate-conscious plastics economy [1,2].

Among the most promising feedstocks for next-generation bioplastics are agricultural residues, lignocellulosic biomass, and food-processing wastes, which represent low-value by-products of existing agro-food systems and therefore constitute “low-impact, zero-burden” carbon sources. These streams are rich in polysaccharides and lignocellulosic fibers that can be hydrolyzed and converted into monomers or directly incorporated as functional fillers, enabling the production of polyesters such as PLA, PHAs, PBS, and starch-based blends with improved mechanical performance and reduced dependence on edible crops. Parallel advances in waste-management hierarchies now prioritise prevention, reuse, high-quality mechanical or chemical recycling, energy recovery in controlled facilities, and landfill only as a last resort, with bioplastics increasingly evaluated for their compatibility with these pathways rather than biodegradability alone [3,4,5].

Within this rapidly evolving landscape, bioplastics have progressed from niche materials to technologically sophisticated polymers deployed in packaging, consumer goods, agriculture, textiles, and automotive components. Recent studies demonstrate that, when sourced from residual biomass and processed in optimised systems, bioplastics can substantially reduce life-cycle greenhouse gas emissions and fossil resource use compared with their petrochemical counterparts. However, trade-offs such as end-of-life greenhouse gas release or unintended microplastic formation must be carefully managed. This review synthesizes current knowledge on feedstock availability, conversion routes, and product performance for biomass-derived bioplastics, with a particular emphasis on the valorization of agricultural and food waste, PHA-based degradation pathways, and modern waste management strategies. By critically assessing technological advances, environmental benefits, and remaining challenges, the article aims to clarify under which conditions bioplastics can deliver genuine sustainability gains and how they can be integrated into resilient, low-carbon material cycles [6,7,8,9,10].

This review critically assesses the contemporary landscape of bioplastics, with emphasis on their feedstock base, conversion routes, and deployment across industrial sectors. It focuses on the valorisation of agricultural residues, lignocellulosic biomass, and food-processing waste as low-impact carbon sources for bioplastic production, highlighting how these streams can underpin more sustainable and circular material flows. In parallel, the article evaluates current plastic-waste management options, including reuse, mechanical and chemical recycling, organic recycling/composting, and energy recovery. It considers how different classes of bioplastics interact with these systems. By synthesizing recent progress, unresolved challenges, and future research opportunities, the review aims to clarify when and how bioplastics can function as credible alternatives to conventional plastics and to identify pathways for integrating them into environmentally responsible, low-carbon product life cycles.

## 2. The Evolution of Bioplastics

**Figure 1:**
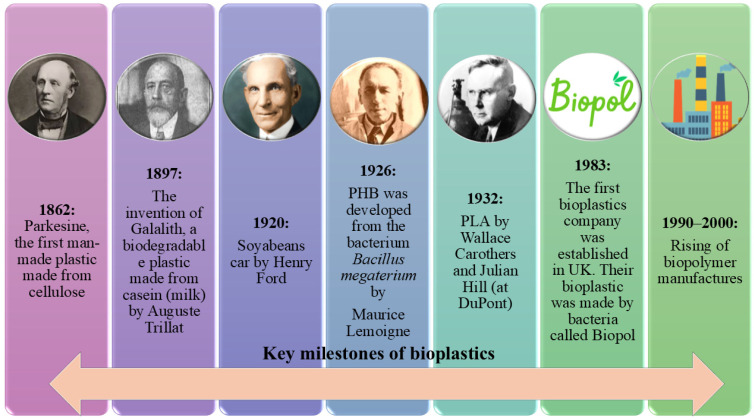
The figure illustrates the key milestones in the history of bioplastics, highlighting significant advancements and discoveries in the field. This timeline highlights the ongoing development of sustainable plastic alternatives.

The evolution of bioplastics has a rich and impactful history, dating back to 1862 when Alexander Parkes introduced Parkesine, the first synthetic plastic derived from cellulose. This pioneering innovation demonstrated the feasibility of using renewable resources for plastic production, laying the foundation for future bio-based polymers. In 1897, Auguste Trillat advanced this concept by developing Galalith, a biodegradable plastic derived from casein (a milk protein), showcasing the potential of natural polymers in material science. The 1920s saw further progress, as Henry Ford pioneered the use of soy-based plastics in automobile manufacturing, highlighting the role of agricultural resources in industrial applications. A breakthrough occurred in 1926 when Maurice Lemoigne discovered polyhydroxybutyrate (PHB), the first microbial bioplastic produced from Bacillus megaterium. This milestone underscored the potential of bacterial fermentation in bioplastic production, a principle that remains central to modern biotechnology. In 1932, Wallace Carothers and Julian Hill at DuPont introduced polylactic acid (PLA), a biobased polymer that remains widely used today due to its biodegradability and versatility. The commercialisation of bioplastics took a significant leap forward in 1983 with the establishment of Marlborough Biopolymers, the first company dedicated exclusively to the production of bioplastics. This venture, a collaboration between Imperial Chemical Industries (UK) and Marlborough Teeside Management, developed Biopol—a bacterial-derived bioplastic based on polyhydroxyalkanoates (PHAs). The successful production and processing of Biopol into strips, filaments, chips, panels, and powders demonstrated the industrial feasibility of microbial biopolymer production, marking the transition from laboratory research to large-scale manufacturing. This breakthrough paved the way for the commercialisation of bioplastics and highlighted the potential of microbial processes in the development of sustainable materials. Between 1990 and 2000, biopolymer production experienced rapid growth, driven by rising environmental concerns, advancements in biotechnology, and increasing demand for eco-friendly alternatives to petroleum-based plastics, as shown in Figure 1. This period saw the emergence of new bioplastics companies, the expansion of production technologies, and the diversification of biopolymer applications across packaging, agriculture, and consumer goods [9].

This era marked a turning point in the bioplastics industry as scientific progress and industrial adoption accelerated. These key milestones reflect the continuous advancements in bioplastics, driving the industry toward more sustainable and innovative solutions for the future [10,11,12].

## 3. The Basics of Bioplastics

Bioplastics refer broadly to polymers derived from renewable biological resources such as plant biomass, agricultural residues, and microbial metabolites. Depending on formulation, these materials may be fully or partially biobased.

The European Bioplastics Association defines bioplastics within three major categories: biobased, biodegradable, or both. This framework aligns with international standards that establish testing protocols and certification criteria for determining renewable carbon content and degradation behaviour. For carbon origin, standards such as EN 16640:2015, EN 16785-1:2015, ISO 16620-4:2016, and ASTM D6866-18 employ radiocarbon (^14^C) and elemental analyses to quantify the renewable fraction within polymers. However, the relationship between origin and degradability is not linear. Specific bio-based polymers, such as bioPE and bioPET, derive from biomass but remain non-biodegradable. In contrast, specific fossil-derived polyesters, such as PBAT, are fully biodegradable and may even meet the composability criteria defined in EN 13432. These exceptions reveal that polymer structure and environmental conditions, rather than feedstock source alone, dictate biodegradability. Hence, accurate labelling and standards-based certification are essential for material validation, consumer communication, and policy enforcement in support of sustainable material transitions [13,14,15,16,17].

### 3.1. Fossil-Based Biodegradable

Petrochemically derived yet biodegradable polymers such as PBAT and PCL exhibit structural characteristics that make them susceptible to enzymatic and microbial attack. These materials serve as promising intermediate solutions for reducing plastic accumulation while maintaining desirable mechanical performance [11].

### 3.2. Biobased Non-Biodegradable

Biobased non-biodegradable or partially degradable plastics, including bioPE, bioPET, bioPP, bioPTT, and bioPA, utilize renewable monomers to reduce fossil dependence while maintaining functional parity with conventional plastics. They represent a key pathway toward sustainable production systems, even though their persistence post-use requires enhanced recycling strategies [12].

### 3.3. Fully Biobased and Biodegradable Polymers

Fully biobased and biodegradable polymers such as PLA, PHAs, and PBS combine renewable feedstock origin with inherent microbial degradability. Their dual environmental advantage positions them as leading candidates for next-generation sustainable packaging, agricultural films, and biomedical applications [7,13].

**Figure 2:**
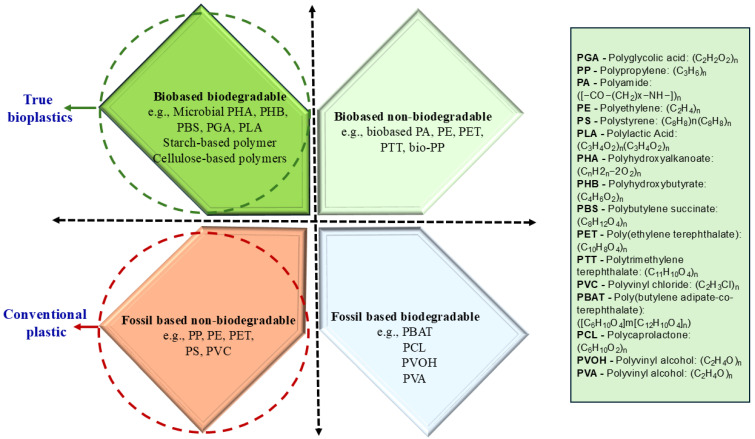
Classification of plastics based on origin (biobased or fossil-based) and biodegradability, illustrating four distinct groups.

Plastics can be systematically categorized into four main groups depending on their carbon origin (biobased or fossil-based) and their capability to biodegrade, as shown in Figure 2. The true bioplastics are both biobased and biodegradable. This group encompasses natural and microbial-derived polymers such as polyhydroxyalkanoates (PHAs), polyhydroxybutyrate (PHB), polybutylene succinate (PBS), polyglycolic acid (PGA), polylactic acid (PLA), and starch- or cellulose-based polymers. These materials originate from renewable biomass and exhibit complete biodegradation under appropriate environmental conditions. The biobased yet non-biodegradable plastics include bio-polyamide (bio-PA), bio-polyethylene (bio-PE), bio-polyethylene terephthalate (bio-PET), bio-polytrimethylene terephthalate (bio-PTT), and bio-polypropylene (bio-PP). While these polymers are produced wholly or partially from renewable biological feedstocks, they retain chemical structures that confer resistance to microbial degradation, thereby offering renewable yet persistent alternatives to conventional plastics. The fossil-based, non-biodegradable polymers include polypropylene (PP), polyethene (PE), polyethene terephthalate (PET), polystyrene (PS), and polyvinyl chloride (PVC). These materials are the most widely utilised synthetic plastics and are also the primary contributors to global plastic pollution, due to their exceptional stability and resistance to natural degradation processes. The fossil-based biodegradable polymers include poly(butylene adipate terephthalate) (PBAT), polycaprolactone (PCL), poly(vinyl alcohol) (PVOH), and poly(vinyl acetate) (PVA). Despite their petrochemical origin, these polymers possess molecular structures that enable microbial and enzymatic breakdown under controlled aerobic or anaerobic conditions, producing environmentally benign end products [14,15].

Bioplastics outperform conventional fossil-based plastics primarily in environmental sustainability metrics rather than mechanical or cost performance. They reduce lifecycle greenhouse gas emissions by 20–70%, with PHA achieving 6.4 kg CO_2_-eq savings per kg compared to PET, due to renewable feedstocks and biodegradability. Unlike persistent petro-plastics that accumulate in landfills (India recycles only 30% of 3.4–26,000 tonnes of daily waste), certified bioplastics like PLA and PBAT enable composting (EN 13432) or anaerobic digestion, diverting 100% of qualifying waste from long-term pollution. Mechanical properties generally lag without modifications. PLA offers comparable tensile strength (50–70 MPa) to PET, but lower impact resistance and heat deflection (55 °C vs. 150 °C), which limits its uses to rigid packaging. Production costs remain 2–4 times higher ($2500–6000/ton vs. $1000–2000/ton for PE/PP), hindering scalability due to limited infrastructure [14,15,16].

## 4. Agricultural Feedstocks for Bioplastics

**Figure 3:**
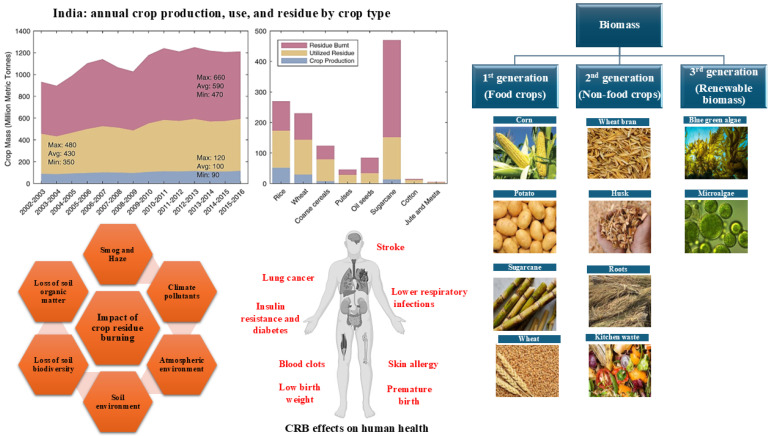
Classification of bioplastic feedstocks into first-, second, and third-generation categories based on their source and sustainability. Adapted with permission from Ref. [15]. This article is an open-access article distributed under the terms and conditions of the Creative Commons Attribution 4.0 International License (https://creativecommons.org/licenses/by/4.0/).

The crop production trends, residue management, human health impacts, and biomass classes suggest that Indian crop residues should shift from open burning to higher-value bioproducts, such as bioplastics, as shown in Figure 3. In the multi-year area chart, the national crop mass is separated into total production, total residue, utilized residue, and residue burnt [15]. The widening difference between residue generated and residue used quantifies a growing surplus of lignocellulosic biomass that is currently dissipated through open-field burning rather than being integrated into resource-recovery chains. The neighbouring bar graph resolves this surplus by crop category, showing that paddy, wheat, and sugarcane dominate residue production and burning, thereby identifying priority feedstocks and geographic hotspots for intervention. This management failure leads to environmental and health burdens. The ecosystem consequences of crop residue burning (CRB) include losses of soil organic matter and biodiversity, degradation of soil structure, and the emission of smoke and climate-relevant pollutants that deteriorate air quality and regional climate [15,17]. Centrally, the human-body silhouette links these emissions to clinical outcomes such as stroke, cardiovascular disease, respiratory infections, lung cancer, insulin resistance, pregnancy complications, and skin disorders, underscoring that residue burning couples agronomic inefficiency with substantial public-health risk and the same agricultural system as a hierarchy of renewable feedstocks for a circular bioeconomy. First-generation (edible) crops, second-generation (non-food residues and organic wastes), and third-generation (algal) biomass are depicted as parallel resource streams capable of supplying carbohydrates, fibres, and lipids for bioplastic production. The crops responsible for large, poorly managed residue flows are simultaneously those that can provide abundant first- and second-generation biomass. At the same time, additional third-generation algal resources can further diversify the feedstock base. Therefore, demonstrates that redirecting crop residues from burning toward bioplastics and other bio-based value chains can simultaneously reduce air pollution and health burdens, improve soil quality, and anchor a rural circular bioeconomy based on sustainable biomass utilisation [18,19,20]. Plant biomass provides an intricate matrix of macromolecules that collectively define the mechanical strength, elasticity, and degradability of all relevant traits for bioplastic applications. Within this framework, cellulose stands as the most abundant renewable polymer on Earth, consisting of β-1,4-linked D-glucose chains organised into crystalline and amorphous domains. These microfibrillar networks impart superior tensile strength to plant cell walls, and when processed into nanofibres or nanocrystals, they act as high-modulus reinforcements in biodegradable polymer matrices [18,19,20].

Hemicelluloses, a structurally diverse group of branched heteropolysaccharides composed of pentose and hexose sugars (e.g., xylans, arabinoxylans, and glucomannans), exhibit amorphous structures and tunable surface chemistries. This versatility enables controlled modification, plasticization, and incorporation into cellulose or starch-based composites to enhance flexibility and barrier performance in biopackaging applications. Lignin, a complex aromatic polymer interlinked through phenylpropanoid units, complements cellulose and hemicellulose by providing rigidity and resistance to microbial degradation in plant tissues. When isolated and chemically modified, lignin can function as a natural compatibilizer, UV screening agent, or reinforcing filler in bioplastic formulations, thereby increasing material durability while maintaining biodegradability. Starch, although commonly recognized as a food and feed polysaccharide, is also a pivotal biopolymer for producing thermoplastics. Under the influence of heat and plasticisers, the native semicrystalline granules of the material undergo gelatinisation, forming thermoplastic starch (TPS) that can be processed using standard polymer technologies. The molecular ratio of amylose (linear chains) to amylopectin (branched chains) dictates the mechanical and retrogradation properties of TPS. Moreover, starch can be blended with agro-industrial by-products, such as potato peels, cereal bran, or fruit residues, to yield cost-effective, biodegradable composites that establish a direct pathway from agricultural waste to high-value biomaterial innovation, as shown in Table 1. Altogether, these lignocellulosic and carbohydrate polymers transform crop residues from an air-polluting burden into a sustainable industrial asset. Their tailored utilisation exemplifies how residue management strategies can advance India’s bioeconomy while mitigating environmental degradation and public health impacts [20,21,22,23,24,25,26,27].

## 5. Production Techniques for Bioplastics

**Figure 4:**
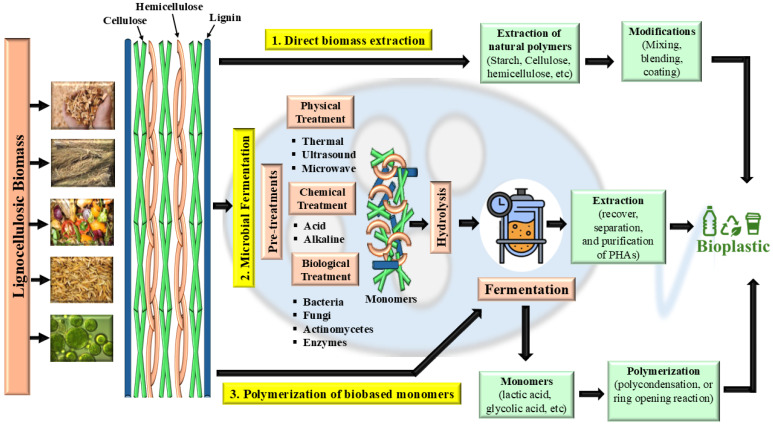
Pathways for bioplastic production from agro-industrial waste, highlighting direct biomass extraction, monomer fermentation, and microbial fermentation routes leading to diverse bioplastic materials.

Recent advancements in bioplastics have explored multiple scientific pathways to synthesise eco-friendly, bio-based plastics. Several industrial-scale methods effectively utilise organic waste streams as raw materials, offering a sustainable approach to bioplastic production. There are three primary methodologies for bioplastic production, as illustrated in Figure 4. Lignocellulosic biomass is converted into bioplastics through three major technological routes.

### 5.1. Direct Biomass Extraction and Modification

In Direct biomass extraction, structural polysaccharides such as starch, cellulose, and hemicellulose are extracted from biomass by mechanical size reduction, solvent or alkaline treatments, and bleaching to remove lignin and impurities. These isolated biopolymers are then modified by mixing with plasticisers, blending with other polymers, or applying coatings to obtain moldable materials that function as starch, cellulose, or hemicellulose-based bioplastics. This approach modifies natural polymers such as starch, cellulose, pullulan, and proteins to create more functional bioplastics. Organic waste rich in these biopolymers is an ideal feedstock due to its abundance, cost-effectiveness, and renewable nature. However, bioplastics obtained through direct extraction often require further enhancements such as blending with plasticizers, incorporating nanoparticles, or undergoing chemical modifications and coatings to improve their mechanical, thermal, and structural properties. A notable breakthrough in this field involved transforming cassava peels into biodegradable bioplastic films using enzymatic catalysis. A specialised enzyme blend, cellulase, xylanase, and glucanase, was employed to hydrolyse the fibrous matrix while preserving the starch molecules. The resulting bioplastic films exhibited impressive mechanical strength and water resistance, rivalling or even surpassing conventional packaging materials. This advancement highlights the potential of waste-derived bioplastics as a sustainable alternative to single-use plastics, contributing to the shift toward eco-friendly materials [31,37].

### 5.2. Polymerisation of Bio-Based Monomers

The production of bioplastics from low-molecular-weight monomers such as lactic acid or glycolic acid derived from the same hydrolysed biomass. These monomers are transformed into high-molecular-weight polyesters (e.g., PLA, PGA, and their copolymers) via polycondensation or ring-opening polymerisation, often using metal or organocatalysts to control molecular weight and stereochemistry. All three routes, which involve the direct extraction and modification of natural polymers, microbial synthesis and extraction of PHAs, and chemical polymerization of fermentation-derived monomers, converge to generate bio-based, potentially biodegradable plastics. The scheme emphasises that effective pretreatment, careful hydrolysis, optimised fermentation, and controlled polymerisation are all essential to convert recalcitrant lignocellulosic biomass into high-performance, sustainable bioplastic materials. The issue identified relates to a mismatch between the stated temperature tolerance target (160 °C) and the reported results, which focus solely on tensile strength improvements with starch loading, without addressing thermal properties. Scientifically, mechanical and thermal properties are distinct performance parameters that should be evaluated together when targeting applications such as food packaging that require specific temperature resistance. However, to achieve thermal resistance targets such as 160 °C, studies recommend additional strategies beyond starch reinforcement, such as blending with other polymers (e.g., polybutylene succinate, PBS), or chemical modifications (e.g., copolymerization) to enhance PLA’s heat deflection temperature and crystallinity [36,37]. Thermal performance is typically assessed by methods like Differential Scanning Calorimetry (DSC) or Heat Deflection Temperature (HDT) testing [38,39,40,41,42,43,44].

### 5.3. Microbial Fermentation-Based Bioplastic

Pretreatments enabling microbial and chemical routes: The physical, chemical, and biological methods used to open the lignocellulosic structure before hydrolysis. Physical pretreatments (thermal, ultrasound, microwave) reduce particle size, disrupt crystallinity, and improve enzyme accessibility, whereas chemical pretreatments (acid or alkaline) solubilize hemicellulose and partially delignify the material; biological pretreatments use bacteria, fungi, actinomycetes, or isolated enzymes to selectively degrade lignin and hemicellulose with lower energy input but longer residence time. Hydrolysis to fermentable monomers: After pretreatment, the carbohydrate fraction undergoes hydrolysis to yield soluble monomeric sugars or other small molecules. Acid or enzymatic hydrolysis converts cellulose and hemicellulose into fermentable hexoses and pentoses that serve as carbon sources for downstream microbial fermentation or as building blocks for chemical synthesis [7].

Microbial fermentation to PHAs: the hydrolysate is fed into a fermenter where selected bacteria convert sugars and other monomers into intracellular polyhydroxyalkanoates (PHAs) under nutrient-limited, carbon-excess conditions. After fermentation, PHAs are recovered from the microbial biomass by cell disruption and solvent or non-solvent extraction, followed by purification to obtain thermoplastic PHA granules that can be processed into bioplastic products. Microbial fermentation is a highly efficient process for producing polyhydroxyalkanoates (PHAs), a key category of bioplastics. This involves culturing microbial strains isolated from natural environments or genetically engineered to optimize PHA synthesis [35,43,45,46]. 

**Figure 5:**
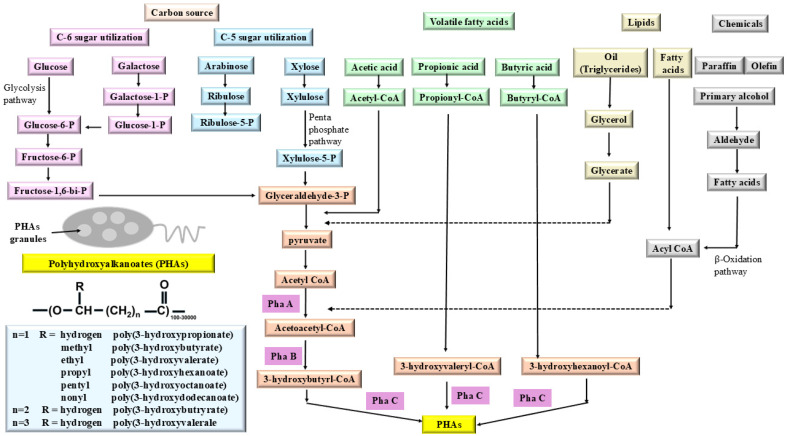
Bioconversion pathways from diverse carbon sources to polyhydroxyalkanoates (PHAs) via central metabolic intermediates and Pha synthase enzymes.

The metabolic routes by which diverse carbon substrates are converted into polyhydroxyalkanoates (PHAs), and the central intermediates, enzymes, and PHA monomer structures in a microbial cell, are shown in Figure 5. The C6 sugars (glucose, galactose) and C5 sugars (arabinose, xylose) enter central metabolism via glycolysis and the pentose phosphate pathway. Glucose and galactose are phosphorylated to glucose-6-P and galactose-1-P, then funnelled through fructose-6-P and fructose-1,6-bisphosphate to glyceraldehyde-3-P and pyruvate. C-5 sugars are converted to ribulose-5-P and xylulose-5-P in the pentose phosphate pathway, which also yields glyceraldehyde-3-P. All these routes converge at pyruvate, which is decarboxylated to acetyl-CoA, the key precursor for PHA synthesis.

The volatile fatty acids (acetic, propionic, butyric acids) and lipid-derived fatty acids are activated to their corresponding acyl-CoA thioesters (acetyl-CoA, propionyl-CoA, butyryl-CoA). Triglyceride oils are hydrolysed to glycerol, which is oxidized to glycerate and then feeds into central metabolism, while free fatty acids and chemically derived fatty acids (from paraffins, olefins, primary alcohols via aldehydes) enter the β-oxidation pathway as acyl-CoA. β-Oxidation shortens acyl-CoA chains, generating acetyl-CoA and related 3-hydroxyacyl-CoA intermediates that can be diverted from energy metabolism toward PHA biosynthesis. In the central lower part, acetyl-CoA is converted to acetoacetyl-CoA by β-ketothiolase, annotated as PhaA. Acetoacetyl-CoA is then reduced to 3-hydroxybutyryl-CoA by acetoacetyl-CoA reductase, labelled PhaB. For medium-chain or copolymeric PHAs, other acyl-CoA precursors (such as 3-hydroxyvaleryl-CoA and 3-hydroxyhexanoyl-CoA) arise from propionyl-CoA, butyryl-CoA, or β-oxidation intermediates. All three 3-hydroxyacyl-CoA monomers are polymerized by PHA synthase, denoted PhaC, to form intracellular PHA granules that appear as inclusion bodies within the cell.

The generic repeating unit of polyhydroxyalkanoates, with the backbone –O–CH–(CH_2_)_n_–C(=O)– and side-chain group R. Variation in R and in chain length parameter n yields different PHA types: when n = 1 and R is hydrogen, the polymer is poly(3-hydroxypropionate); when n = 1 and R is methyl, it is poly(3-hydroxybutyrate), and larger alkyl groups (ethyl, propyl, pentyl, nonyl) correspond to longer-chain PHAs like poly(3-hydroxyvalerate), poly(3-hydroxyhexanoate), or poly(3-hydroxynonanoate). Copolymers (e.g., poly(3-hydroxybutyrate-co-3-co-3-hydroxyvalerate)) arise when different 3-hydroxyacyl-CoA species are incorporated into the same polymer chain, altering thermal and mechanical properties. Overall, the figure shown in Figure 5 emphasizes that PHAs are metabolic storage polymers synthesized by microorganisms from a wide range of carbon sources, including simple sugars, volatile fatty acids, oils, and chemically derived fatty acids. It integrates central carbon metabolism, lipid catabolism, and specific PHA biosynthetic enzymes (PhaA, PhaB, PhaC) to illustrate how environmental carbon availability and intracellular acyl-CoA pools determine the composition and structure of PHA granules [47,48].

## 6. Applications for Bioplastics

Biodegradable plastics offer an effective alternative to conventional plastics, particularly in applications where composting is recommended, such as mulch films with high organic content or scenarios where waste collection and treatment infrastructure are limited. It is crucial to differentiate between biodegradation and composting: biodegradation refers to the natural breakdown of materials by microorganisms over an undetermined time frame and under various environmental conditions, whereas composting is a controlled biological process requiring specific conditions (e.g., temperature, moisture, aeration) to convert materials into nutrient-rich compost within a defined time period, usually 90 to 180 days in industrial facilities. All compostable plastics are biodegradable, but not all biodegradable plastics meet the stringent standards required to be certified as compostable, which prevents the formation of harmful residues and ensures complete degradation [45].

Globally, the production of 100% bio-based polymers is approximately 2 million tonnes per year, with biodegradable plastics accounting for about two-thirds of this total. The bioplastics sector in Europe is growing at an estimated annual rate of 10%, driven by regulatory policies and consumer demand for sustainable alternatives. If bioplastics received subsidies and political support like those for biofuels, their global growth could accelerate by 10–20%. The packaging sector dominates bioplastic consumption, accounting for 48% of bioplastic usage in 2022. Its applications include films, bags, containers, disposable products (such as razors, utensils, diapers, and feminine hygiene products), as well as cosmetic and personal care packaging. Emerging materials, such as polylactic acid (PLA), offer chemical recycling advantages through hydrolysis, allowing for the recovery of monomers for reuse. Moreover, algae-derived biopolymers show promise in diverse fields from biodiesel production to pharmaceutical applications, including antibacterial and anticancer uses. Bioplastics combine lightweight, strength, and durability qualities, making them well-suited for everyday products as global plastic demand continues to rise. This emphasizes their key role in reducing environmental impact and supporting sustainable development, as shown in Table 2. A critical ecological challenge is the vast quantity of agricultural waste generated annually. Traditional disposal methods, such as open burning and landfilling, cause pollution and squander valuable biomass resources. Utilizing agrarian waste as a raw material for industrial bioplastic feedstock not only mitigates waste management issues but also provides a sustainable, cost-effective resource. This approach supports circular economy principles by repurposing biomass residues into high-value materials, enhancing both environmental and economic sustainability [49,50].

**Figure 6:**
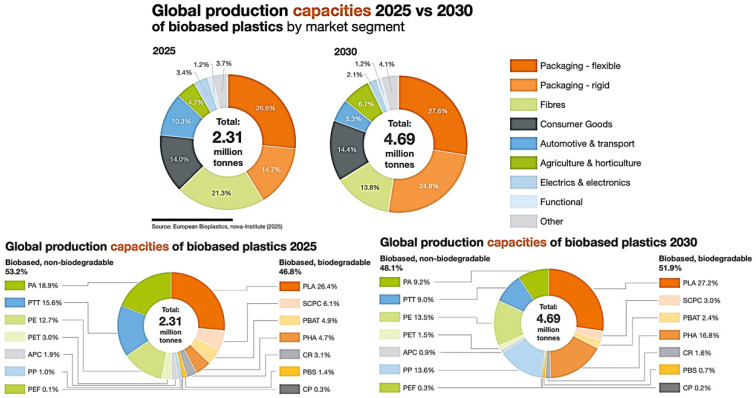
Projected Global Production Capacities of Biobased Plastics by Market Segment and Polymer Class, “2025–2030”.

In 2025, the worldwide installed capacity for biobased plastics is approximately 2.31 million tonnes, rising to 4.69 million tonnes by 2030, representing an approximate doubling over five years. Packaging is the principal sink in both years: flexible packaging alone accounts for roughly one-quarter of capacity, followed by rigid packaging. Meanwhile, fibres, consumer goods, automotive and transport, agriculture and horticulture, electrical and electronic components, functional materials, and miscellaneous “other” uses share the remaining fraction. The relative shares of these sectors remain broadly similar between 2025 and 2030, indicating that the capacity increase is primarily a scaling up of existing applications rather than a redistribution among industries. The production capacities of biobased plastics in 2025 versus 2030, resolving both end-use sectors and polymer classes, and distinguishing biobased–biodegradable from biobased–non–biodegradable materials are shown in Figure 6 [51,52].

In 2025, non-biodegradable biobased polymers (notably bio-PA, PTT, bio-PE, bio-PET, and PEF) constitute approximately 53% of the total capacity, reflecting their strong use in durable applications such as fibers and engineering plastics. By 2030, this share decreases to roughly 48%, indicating a modest but measurable shift toward biodegradable systems as capacity expands. Among biodegradable bioplastics, PLA is the leading polymer in both years, representing roughly a quarter of all biobased plastic capacity in 2025 and a slightly larger fraction in 2030, consistent with its mature technology and broad use in packaging and fibres. Starch-containing polymer compounds (SCPCs), PBAT-based blends, PHA, PBS, and other compostable polyesters (“CR”, “CP”) provide minor but significant contributions, with PHA showing strong relative growth by 2030, reflecting industrial upscaling of microbial routes. The bio-PA, PTT, and bio-PE are the main contributors in 2025; their absolute capacities are expected to increase by 2030, but their percentage shares will fall slightly because the biodegradable segments are projected to grow faster. Overall, a transition toward higher global tonnage of biobased plastics, with a gradually increasing fraction of biodegradable chemistries, is expected, while packaging remains the key market driver [51,52,53,54].

**Table 2:** Mechanical performance and practical uses of various biopolymer materials.

Biopolymer Used	Mechanical Properties	Applications	References
PBSA	Thickness 800 μm Tensile strength, 21.0–22.0, 14.1–17.3, 12.3–16.1	Mulching film	[52]
Chitosan	Thickness, 53–86 μm Tensile strength, 0.68–2.92 MPa	Pharmacy Food packaging	[53]
Soy protein	Tensile strength, 4.3–5.7 MPa	Food packaging	[54]
Orange peel pectin	Thickness, 146–199 μm Tensile strength, 5–9.6 MPa Tensile	Heat-sealable pouch Active food coating	[55]
Genetically modified flax fiber	-	Wound dressing	[56]
Cashew nutshell starch-walnut shell cellulose	-	Acting packing	[57]
Chitosan nanoparticles (CSNPs)	Tensile strength 46.19 ± 2.31 MPa	antioxidant and antibacterial additives for active bioplastic packaging	[58]
Cellulose	Tensile strength 95 MPa	Active food packaging	[59]
PBS	-	Food packaging	[60]
Orange fiber residue	Tensile strength 3.52 MPa	Mulching film	[61]

## 7. Bioplastic Life Cycle Alignment with Natural Carbon Turnover

Recent research increasingly positions agricultural biomass waste as a “low-impact, zero-burden” feedstock for renewable materials, because it is generated as a by-product of existing food and fiber production rather than requiring additional land, water, or agrochemicals. In contrast to conventional agriculture, which valorizes only grain, fruit, or fiber, a circular bioeconomy aims to mobilize the entire plant system by converting residues such as stalks, leaves, husks, shells, and pruning wastes into fermentable sugars, fibers, and bio-oils that can serve as precursors for bioplastics and other bio-based chemicals. This paradigm shift reduces open field burning and uncontrolled decomposition of residues, thereby lowering particulate emissions and greenhouse gas release, while adding value streams for farmers. Within this context, industrial hemp has emerged as a particularly attractive multipurpose crop. Its high biomass productivity, deep root system, and relatively low demand for pesticides and fertilizers make it suitable for less fertile soils and cooler climates. Meanwhile, its anatomical fractions, including cellulose-rich bast fibres, lignocellulosic hurds, and oil-containing seeds, can simultaneously feed the textile, construction material, bio-composite, and polymer precursor markets. When such biomass is directed toward bioplastic production, either as a direct fiber reinforcement or as a carbohydrate source for fermentative monomer synthesis, it enables the production of materials with a substantially reduced fossil carbon footprint. Bioplastics derived from these residues, especially when designed for biodegradability or effective recyclability, therefore represent a key technology for coupling waste valorization, rural income generation, and mitigation of plastic pollution within a scientifically grounded framework of sustainable development [62].

**Figure 7:**
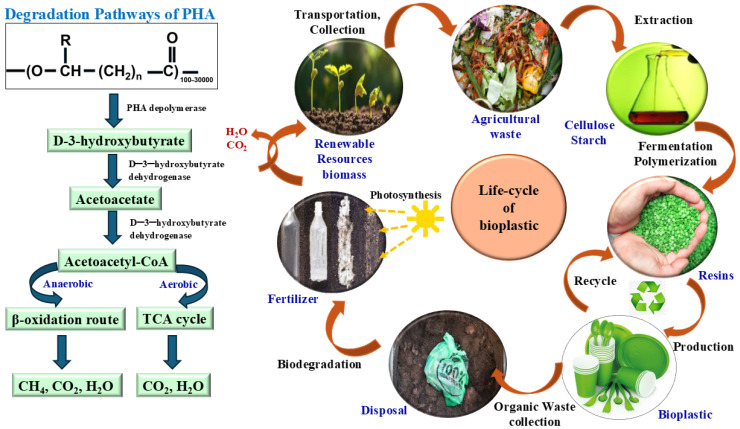
Integrated Life Cycle and Biodegradation Pathways of PHA-Based Bioplastics.

The degradation pathway of polyhydroxyalkanoate (PHA) bioplastics, along with the system-level life cycle of bioplastic products, as shown in Figure 7, highlights how both operate as coupled loops within a circular bioeconomy. At the biochemical level, intracellular or environmental enzymes depolymerize PHA into D-3-hydroxybutyrate, which is oxidized to acetoacetate and converted to acetoacetyl-CoA; this central intermediate is then funnelled either into the tricarboxylic acid cycle under aerobic conditions, yielding CO_2_ and H_2_O, or into anaerobic β-oxidation-like and methanogenic pathways, producing CH_4_, CO_2_, and H_2_O. In parallel, the macroscopic life cycle shows agricultural residues being transformed via extraction, fermentation, and polymerization into bioplastic resins and finished products, which, after use, enter organic-waste streams where the same microbial processes drive their mineralization to CO_2_, H_2_O, and nutrient-rich organic matter that returns to soil as fertilizer and supports new biomass through photosynthesis. Comparing the two, the degradation pathway represents the intrinsic metabolic compatibility of PHA with microbial central carbon metabolism, while the right-hand life-cycle loop represents the extrinsic technospheric cycle in which bioplastics serve as temporary carbon reservoirs before re-entering biogeochemical cycles; together they illustrate that, unlike conventional petro-plastics, biodegradable bioplastics can be designed so that their product life cycle and molecular degradation route are mechanistically aligned with natural carbon turnover rather than in conflict with it [55,56,57].

Bioplastics offer diverse pathways, including mechanical/chemical recycling (bio-PE/PET, compatible with conventional streams), industrial composting (PLA/PBAT, as per EN 13432, yielding compost from organic waste), and anaerobic digestion for biogas production. Landfilling risks microplastic persistence if non-certified; unlike the dominance of incineration/recycling for fossil plastics, biodegradability diverts waste but requires segregated infrastructure. India’s low recycling rates amplify the need for certified EOL systems, and Upfront costs burden bioplastics, but LCAs reveal 20–70% lower GHG emissions (e.g., PHA saves 6.4 kg CO_2_-eq/kg vs. PET) and reduced pollution externalities. Long-term savings emerge via avoided remediation and circular recovery, positioning bioplastics competitively with subsidies. In India, residue-to-bioplastic chains could enhance viability [14,63,64].

## 8. Global Portfolio of Commercial Bioplastics

Bioplastic production costs range from $2500–$6000 per ton, 2–4 times higher than conventional plastics ($1000–$2000 per ton for PE/PP), driven by limited scale and feedstock variability. Global capacity is projected to reach 2.31 million tonnes in 2025, increasing to 4.69 million tonnes by 2030, which could potentially reduce costs through economies of scale. In India, bioplastics remain niche amid 3.4–26,000 tonnes of daily plastic waste, with 30% recycling, though policy incentives like EPR could bridge the gap [51].

The distinct classes of bioplastics have progressed from laboratory systems to established commercial materials, each occupying a clearly defined techno-economic niche, as shown in Table 3. Bio-based aliphatic polyesters, such as PLA and PBS (e.g., Ingeo, Luminy, Bionolle), now serve high-volume packaging markets, offering both rigid and flexible formats, as well as hot-fill and capsule applications, and agricultural mulch films, while meeting industrial composability criteria when properly formulated. Starch-rich formulations (Mater-Bi) and advanced starch/PLA/PBAT blends (Bio-Flex, ecovio) demonstrate the upgrading of native polysaccharides into film and bag grades with controlled disintegration in organic recycling infrastructures, as well as compatibility with conventional film processing equipment.

The portfolio further demonstrates the functional breadth of bioplastics through microbially produced PHAs (Mirel, Nodax), which are designed for rapid biodegradation in soil and aquatic environments and are therefore suited to products with a high risk of environmental leakage, such as straws, coated paper, and other single-use items. At the opposite end of the degradability spectrum, drop-in bio-polyolefins, such as I’m Green bio-PE, retain the durability and recyclability of conventional polyethylene while substituting bio-derived carbon for fossil feedstocks in bottles, caps, films, and consumer goods. Collectively, these commercial examples underscore that bioplastics constitute a continuum of material solutions, ranging from non-biodegradable but bio-sourced polymers to fully biodegradable polyesters, whose molecular design and product engineering are increasingly tailored to align with evolving environmental and regulatory requirements, as well as performance and processing [64,65].

## 9. Challenges and Future Directions

Biodegradable, agriculture-derived plastics face several interlinked challenges. Technically, many bioplastics still have narrower processing windows, lower heat distortion temperatures, or inferior barrier and mechanical properties compared with benchmark petro-plastics, limiting their use in high-performance applications. Economically, feedstock logistics, pretreatment, and downstream purification remain cost-intensive, and bioplastics often compete with heavily optimized, large-scale petrochemical processes. Environmentally, actual end-of-life benefits depend on access to industrial composting or appropriate recycling systems; mismanaged bioplastics can still contribute to litter and microplastic formation, especially when biodegradation conditions are not met. Finally, there is persistent public confusion between “bio-based,” “biodegradable,” and “compostable,” which can drive inappropriate disposal and undermine environmental gains. Future directions focus on overcoming these constraints through integrated innovation. At the material level, metabolic engineering and catalyst design should yield new copolymers, blends, and nanocomposites that match or surpass conventional plastics in performance while maintaining controlled biodegradability. Process-wise, embedding bioplastic production into lignocellulosic and algal biorefineries, with energy and water integration, can reduce costs and carbon footprints. On the system side, harmonized global standards for biodegradation and composability, more transparent labelling, and expanded infrastructure for organic waste collection, advanced recycling, and industrial composting are critical. Coupling these developments with robust life-cycle assessment, policy incentives, and circular design principles will ensure that agricultural bioplastics evolve from niche alternatives to core components of a sustainable, low-carbon plastics economy [58,59,75].

## 10. Conclusions

The collective utilization of agriculture-derived feedstocks, encompassing first-generation crops, second-generation residues, and third-generation algal biomass, establishes a sustainable and renewable carbon base pivotal for next-generation bioplastics. Through advanced pretreatment, hydrolysis, microbial fermentation, and precision polymerization, these pathways enable the transformation of lignocellulosic biomass into structurally versatile polymers such as PLA, PHAs, and PBS. These bioplastics demonstrate competitive performance with controlled biodegradability, yielding significantly reduced greenhouse gas emissions 20% to 70% lower than conventional fossil-based plastics, thereby aligning material science with global climate mitigation goals. Biocomposites derived from lignocellulosic residues represent a critical innovation frontier, offering enhanced mechanical properties, including increased tensile strength and reduced moisture sensitivity, overcoming inherent limitations of pure bioplastics. The integration of natural fibers such as hemp and crop residues into bioplastic matrices promotes local valorization of abundantly available agricultural by-products, contributing to cost-effective, scalable bio-based solutions. Such hybrids not only improve material performance but also embed circularity at the molecular and macroscopic levels, enabling product lifecycles that harmonize with natural carbon cycles and biodegradation kinetics. To fully realize these benefits, strategic prioritization of non-edible biomass feedstocks will minimize food–feedstock competition while advancing integrated biorefinery concepts that optimize sugar yields and resource efficiency. The anticipated economic and environmental benefits include significant reductions in plastic pollution, improved soil quality, greenhouse gas mitigation, and the creation of rural bioeconomy livelihoods. This review underscores bioplastics and biocomposites not merely as alternatives but as transformative agents in the transition from linear, fossil-dependent plastics to a circular bioeconomy. Their development is underpinned by multidisciplinary integration of agricultural biotechnology, materials engineering, and circular economy principles, positioning them as pivotal materials for sustainable industrialization, climate resilience, and inclusive economic growth.

## Figures and Tables

**Table 1:** Agro-industrial feedstocks and their contributions to bioplastic production.

Agro-Industrial Feedstocks	Types of Bioplastics Produced	Pre-Treatment	Plasticizers	Innovation	References
Corn, banana, and potato peels	Starch-based bioplastic	HCl solution	Glycerol	Decomposition within 28 days	[21]
Banana peel	Starch, pectin, and chitosan blend-based bioplastic	Acidic (Hydrochloric acid)	Glycerol	After 3 days, (97 ± 2.5%) biodegradation	[22]
Hemp stalk waste	xylan (hemicellulose) based bioplastic	Citric acid (CA) and dicumyl peroxide	PEG, glycerol	Works as an electrical Insulator with an elastic modulus of up to 2.9 GPa, tensile strength reaching 70 MPa, and elongation up to 4.3%	[25]
Cocoa pod husk and sugarcane bagasse waste	Cellulose and fibre-based bioplastic	Alkaline (NaOH)	Glycerol and sorbitol	Used in food packaging	[27]
Corncob residues	Cellulose and lignin-based bioplastic	Ethanol	Metal salt solution	Recyclable, biodegradable, and water stable, with mechanical properties of 136 MPa	[28]
Flax fibre and cotton linters	Cellulose acetate-based bioplastic	Acidic (Acetic anhydride, glacial acetic acid, and sulphuric acid)	Polyethylene glycol 600	After 14 days, 41–44% biodegradation	[29]
*Moringa oleifera* gum (MOG)	plant gum-based bioplastic	Thermal (50 °C for 5 h)	poly (vinyl alcohol) (PVA), glycerol (Gly, plasticizer), and citric acid (CA, cross-linker)	Diverse physicochemical properties, from hydrophilicity to hydrophobicity and rigidity to flexibility	[30]
Rice bran	Starch and protein-based bioplastic	Hexane	Glycerol	decomposition within 30 days under composting conditions	[31]
Tea waste	cellulose, hemicellulose, lignin, and protein-based bioplastic	Citric acid	-	Tensile strength of 6.16 MPa and an elongation at break of 13.33%	[32]
Cassava peel waste	cellulase, xylanase, and glucanase-based bioplastic	enzyme blend (0.7% cellulase, 0.3% xylanase, 0.5% β-glucanase)	Glycerol	Commercial packaging materials	[33]
Wheat straw	Lignocellulosic-based bioplastic	Alkaline and urea	-	High tensile strength, 101.78 MPa, water stability, thermal stability, and UV resistance	[34]
Tofu liquid waste	carboxy methyl cellulose (CMC) based bioplastic	-	Sorbitol	Robust properties: thickness of 0.110 mm, a water vapour permeability rate of 0.584 g m^−2^h^−1^, a tensile strength of 146.3 kg/cm^2^, and an elongation of 97.04%	[35]
Cottonseed meal	Protein-based bioplastic	NaOH solution	Glycerol	Used as plantable pots/containers in agriculture and horticulture	[36]
Citrus peel waste	Cellulose and pectin-based bioplastic	Citric acid and ultrasonic	-	Excellent flexibility, water stability, gas barrier properties, and antimicrobial functionality significantly extend shelf life when used as packaging.	[37]

**Table 3:** Overview of Commercial Bioplastics: Brands, Producers, Origins, Launch Years, and Applications.

Bioplastic Nature/Type	Brand Name	Company Name	Country	Typical Uses	References
Starch-based, biodegradable blend	Mater-Bi	Novamont S.p.A.	Italy	Compostable carrier bags, organic-waste liners, agricultural mulch films, and food-service items.	[65]
Biobased, biodegradable PLA	Ingeo	NatureWorks LLC	USA (large plants in USA/Thailand)	Rigid and flexible packaging, disposable tableware, fibers, and 3D-printing filament.	[66]
Biobased, biodegradable starch/PLA/PBAT blends	Bio-Flex	FKuR Kunststoff GmbH	Germany	Flexible packaging films, mulch films, and disposable tableware.	[67]
PLA (Polylactic Acid)	BioYug	Balrampur Chini Mills Ltd.	India	Compostable bottles, films, packaging	[68]
PLA & Bio-compostable	Biopac	Biopac India	India	Cups, plates, trays, containers	[69]
Biodegradable/Compostable (PLA-based)	Ecolastic	Ecolastic Products Pvt. Ltd.	India	Single-use items, packaging, and defense products	[70]
Biobased, partially biodegradable polyester (bio-PE)	I’m green bio-PE	Braskem	Brazil	Drop-in polyethylene for bottles, caps, films, and consumer packaging; not biodegradable but biobased.	[71]
Partly bio-based, biodegradable blend (PLA + ecoflex and others)	ecovio	BASF SE	Germany	Compostable shopping bags, organic-waste bags, coffee cups, agricultural films, and coated paper.	[72]
Fossil-based, biodegradable aliphatic–aromatic polyester (PBAT)	ecoflex	BASF SE	Germany	Flexible films, compostable bags, and blend components to increase the toughness of PLA or starch plastics.	[73]
Biobased, biodegradable PLA	Luminy	Total Corbion PLA	The Netherlands/Thailand	Food packaging, hot-fill cups, fibers, 3D-printing, and technical parts.	[74]

## Data Availability

No new data were created or analyzed in this study. Data sharing is not applicable to this article.
